# Effects of early life and current housing on sensitivity to reward loss in a successive negative contrast test in pigs

**DOI:** 10.1007/s10071-019-01322-w

**Published:** 2019-11-13

**Authors:** L. Luo, I. Reimert, E. A. M. Graat, S. Smeets, B. Kemp, J. E. Bolhuis

**Affiliations:** Adaptation Physiology Group, Department of Animal Sciences, Wageningen University and Research, PO Box 338, 6700 AH Wageningen, The Netherlands

**Keywords:** Pigs, Enrichment, Early life, Reward loss, Coping style, Affective state

## Abstract

Animals in a negative affective state seem to be more sensitive to reward loss, i.e. an unexpected decrease in reward size. The aim of this study was to investigate whether early-life and current enriched vs. barren housing conditions affect the sensitivity to reward loss in pigs using a successive negative contrast test. Pigs (*n *= 64 from 32 pens) were housed in barren or enriched conditions from birth onwards, and at 7 weeks of age experienced either a switch in housing conditions (from barren to enriched or vice versa) or not. Allotting pigs to the different treatments was balanced for coping style (proactive vs. reactive). One pig per pen was trained to run for a large reward and one for a small reward. Reward loss was introduced for pigs receiving the large reward after 11 days (reward downshift), i.e. from then onwards, they received the small reward. Pigs housed in barren conditions throughout life generally had a lower probability and higher latency to get the reward than other pigs. Proactive pigs ran overall slower than reactive pigs. After the reward downshift, all pigs ran slower. Nevertheless, reward downshift increased the latency and reduced the probability to get to the reward, but only in pigs exposed to barren conditions in early life, which thus were more sensitive to reward loss than pigs from enriched early life housing. In conclusion, barren housed pigs seemed overall less motivated for the reward, and early life housing conditions had long-term effects on the sensitivity to reward loss.

## Introduction

The barren housing conditions in which most commercial pigs world-wide are housed limit the expression of important species-specific behaviours, like foraging and exploration (De Jonge et al. 1996; Studnitz et al. 2007; Wemelsfelder et al. 2000), and thereby increase the frequency of manipulative behaviours directed at pen mates, such as ear and tail biting (Beattie et al. 2000; Bolhuis et al. 2006; Carreras et al. 2016). Pigs in barren housing, moreover, show physiological signs of chronic stress (Beattie et al. 2000; Bolhuis et al. 2006; Carreras et al. 2016). This chronic stress could also be linked to a psychological state of (mild) depression. Indeed, pigs housed in barren conditions have been found to show a more pessimistic response in a judgement bias task, suggesting that they have a more negative affective state, compared with pigs in enriched conditions (Douglas et al. 2012), although in another study, no such effect of housing was found (Carreras et al. 2016).

A negative affective state may also enhance the sensitivity to reward loss (Chaby et al. 2013). For instance, people in a state of depression have been shown to be more susceptible to loss or failure (Tucker and Luu 2007). The response to reward loss can be measured in animals by a successive negative contrast (SNC) test (Burman et al. 2008; Flaherty et al. 1998). In a SNC test, reward loss is induced by unexpectedly decreasing the reward size or quality for animals that have been trained. This may induce a transient, potentially ‘disappointment-like’, aversive affective state, caused by the discrepancy between the anticipated reward, i.e. the reward the animals expected to receive, and the actual reward (Justel et al. 2014; Papini 2014; Rosas et al. 2007). How aversively the animals respond to such a discrepancy (e.g., depending on the task, by reduced operant responses or a slower speed to get to the reward), has been suggested to be a sign of the animals’ background, longer-term, affective state or mood (Flaherty et al. 1998; Mitchell et al. 2012; Riemer et al. 2016). In line with this, it has been shown that rats experiencing removal of enrichment in their home cages, thereby likely having a more negative affective state, responded stronger to a reward downshift, compared to control rats (Burman et al. 2008; Chaby et al. 2013). Increased sensitivity to reward loss appears thus to reflect negative affective states in humans and animals, and it could, therefore, be an useful measure of such states in pigs as well, which has, to the best of our knowledge, not been studied in this species so far.

In the current study, we do not only address the impact of housing conditions under which pigs are kept on their affective state, but also the influence of a barren vs. enriched environment in early life. It has been shown that adverse conditions in early life can have long-term effects on behaviour, physiology, and cognition. For example, isolation in early life decreases the motivation for social contact and sucrose-drinking in later life in rats (Van den Berg et al. 1999). Also in pigs, long-lasting effects of early life experiences have been found (Telkänranta and Edwards 2017). Importantly, pigs that changed from enriched housing in early life to barren housing later on show as much signs of decreased welfare as pigs kept in a barren environment throughout life, or even more (Bolhuis et al. 2004; Munsterhjelm et al. 2009), which suggests that a loss of enrichment in later life could be even more detrimental than barren housing throughout. In line with this, barren housed pigs tended to show more pessimistic judgement biases after they had temporarily experienced an enriched environment (Douglas et al. 2012). Thus, the impact of housing on affective states may not only depend on the actual housing conditions, but also on the conditions present in early life, and these two may interact.

The aim of this study was, therefore, to investigate the combined effects of early life environment and current housing conditions on the sensitivity to reward loss in pigs. Hereto, pigs housed in barren or enriched conditions in early life and experiencing either a switch in housing conditions at 7 weeks of age or not, were subjected to a SNC test at 12 weeks of age in which they had to run a U-shaped track to get to a food reward. Downsizing of the reward was expected to slow down the latency to get to the reward, indicative of an experience of reward loss. We hypothesised that barren housed pigs, and particularly those that had experienced enrichment in early life, would be more sensitive to reward loss, as they were expected to have a more negative affective state.

Personality traits may have an effect on behavioural responses in test situations, including negative contrast (Cuenya et al. 2012), attention bias (Franklin et al. 2016) and judgement bias tests (Asher et al. 2016). Therefore, pigs allotted to the different housing and rearing conditions in this study were balanced for their coping style, i.e. a personality trait related to the way an individual copes with challenging situations, as assessed in a backtest in early life (Hessing et al. 1994; Reimert et al. 2014b).

## Materials and methods

The established principles of laboratory animal use and care were followed, as well as the Dutch law on animal experiments. The Animal Care and Use Committee of Wageningen University & Research approved the experiment.

### Animals and housing

In this experiment, 64 pigs (Tempo × Topigs *20*) from 29 sows, equally divided over 2 batches were studied. Sows were inseminated on the same day in each batch, and were housed in Carus, the animal facilities of Wageningen University & Research, Wageningen, the Netherlands, from 1 month before farrowing. From birth till weaning (around 28 days of age), half of the piglets within each batch were housed in 8.6 m^2^ barren (B1) pens with a solid floor and a small area with slats. The other half were housed in 17.1 m^2^ enriched (E1) pens with the same farrowing part (8.6 m^2^) as the barren pens, and with an additional enriched part. In the enriched part, 1.7 kg straw, 300 L sawdust, and 270 L peat were provided as substrates. Besides, 0.8 kg straw and 40 L of sawdust were added daily, and 30 L of peat was added weekly in the enriched pens. Additionally, two objects for manipulation, one chain with a ball and one chain with screws that touched the floor were placed in the barren pens. Two objects, one chain with a ball and an object that was alternated daily and selected from four different ones, were placed in the enriched pens. All sows were housed in the same farrowing crates without access to the enrichment. In the first week after birth, one heating lamp was provided in the barren pens, and two lamps in the enriched pens. Each pen had one drinking nipple for the piglets and one for the sow. Sows were fed a standard commercial diet twice a day. From 5 days of age onwards, piglets received some creep feed. Temperature was set at 25 °C at birth, and gradually decreased to 21 °C over the course of 2 weeks. Each pen was cleaned daily, and lights and a radio were on from 7:00 until 19:00 h.

At an average of 28 days of age, pigs were weaned and 192 pigs (96 per batch) were selected and regrouped in 32 new pens, which contained 6 healthy non-littermate pigs (from the same pre-weaning treatment) each. Per group, 3 males and 3 females from different litters were selected, balanced for coping style (3 HR and 3 LR, assessed and classified as described in Reimert et al. 2014a). Pigs fulfilling the criteria with a body weight closest to the litter mean weight were preferably selected so that the average body weight of the post-weaning selection was representative of that of the whole group at weaning. Housing treatment (B1 vs. E1) for each pig was kept the same as before weaning. After weaning, half of the pigs were housed in 5.6 m^2^ barren pens, with a partly solid and slatted floor. The other half of the pigs were housed in 11.2 m^2^ pens enriched with 2.5 kg straw, 400 L of sawdust, and 360 L of peat on the floor. Additionally, 1.25 kg straw and 60 L sawdust were added daily, and 45 L peat was added weekly in the enriched pens. The toys in the barren and enriched pens were kept the same as before weaning, and from 39 days of age, enriched housed pigs received extra enrichment such as a jute sack, a rope, branches or an egg tray on each Monday until the end of the experiment (day 133).

Each pen had one drinking nipple and pigs received solid food ad libitum. On the weaning day, the temperature was set at 25 °C and it was gradually decreased to 21 °C over the course of 2 weeks and kept until the end of the experiment. After weaning, one heating lamp was provided in each pen for the duration of 2 weeks. Lights and a radio were on from 7:00 until 19:00 h.

At an average of 47 days of age, half of the pigs experienced a switch in housing conditions, resulting in four treatment groups, E1E2, E1B2, B1E2, B1B2, *n *= 8 pens per group. For this switch, they were moved to a different pen. B1B2 and E1E2 groups were also removed to new pens, but without a change in housing condition. After this switch, straw, peat and toys were used and added in amounts as described before, but only 30 L of sawdust was added daily in the enriched pens.

### Successive negative contrast test

To assess the sensitivity for reward loss as a measure of affective state, a successive negative contrast (SNC) test was applied from 84 to 120 days of age. Two female pigs per pen (one LR and one HR), in total 64 pigs, were selected from the 32 pens. These 64 pigs were 60 females and 4 males (of the 64 female pigs we initially selected, four had health issues. We replaced those with males from the same pen having the same coping style from the beginning of the test. All four males were from different pens, and 1 from B1B2, two from E1B2, and 1 from B1E2 housing conditions). Before the test period started, all pigs received pieces of apple in their home pen to accustom them to the reward in the test. Pieces of apple have been successfully used as a reward in previous research, and it has been shown that pigs prefer multiple pieces of apple (4, large reward) over a single piece (Melotti et al. 2013). Pigs were food deprived for at least 1 h before the test, and allowed to eat after the two pigs in a pen finished the test.

**Fig. 1:**
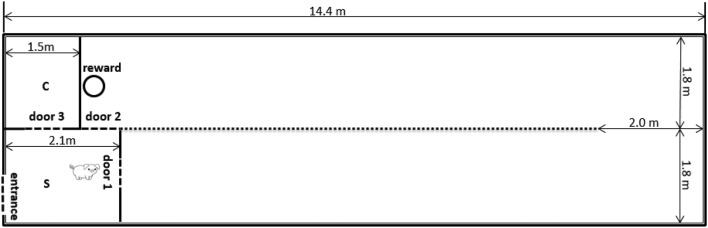
Layout of the test area used for the successive negative contrast test. The area consisted of a U-shaped runway. The two arms of the runway (12.3 × 1.8 m and 12.9 × 1.8 m) were separated by a wired fence (height: 1.2 m). Pigs were brought in a start box (S) and entered the runway from door 1. Pigs could run in the U-shaped runway to get the reward in a plastic plate (diameter 32 cm) screwed on the floor. After pigs finished a trial, they left the runway from door 2 and waited in the start box for another trial, until all three trials were finished. Two experimenters stayed in a compartment (C) to operate the doors and record the latencies

The test area was in a separate room away from the home pens. Pigs were habituated to the test arena in 3 days with three trials per day, initially in pairs (trial 1 on day 1), thereafter individually with a pen mate waiting in the start box (trial 2 and 3 on day 1), and finally individually without the presence of a pen mate (days 2 and 3). After this habituation period, pigs were individually allowed to run from a start box down a U-shaped runway (Fig. 1) to obtain a food reward from a round plastic plate (diameter 32 cm) at the end. One pig per pen received six pieces of apple (1 piece was 1/48 part of an apple (Elstar variety, without the core) as the large reward on each trial, and the other one received 1 piece of apple as the small reward. Even though all pigs ate apple in their home pen, some pigs (*n *= 6) did not eat apple in the test and were given banana instead (1 piece was 1/4 of a 1 cm-thick slice) after the habituation days. After habituation, pigs were allowed to run for the food reward on 4 days per week. Each pig did three trials per day, with a maximum of 120 s per trial. A trial was finished when the pig ate the reward within the 120 s, or when the pig turned back five times (i.e. started walking in a direction not leading to the reward) or, in case the pig had not finished eating the reward within the maximum time set, at 120 s. During each trial, the latency to leave the start box (i.e. the pig was in the runway with all 4 legs) and the latency to start eating were recorded. From these two latencies the latency to reward was calculated by subtracting the latency leaving the start box from the latency to start eating. If a pig did not start eating the reward within the 120 s, the latency to reward was set to 120 s.

Pigs were excluded from further testing if they reached the maximum time (120 s) and did not reach the reward on three consecutive test days (*n *= 7 pigs), if they reached the reward but did not eat it on three consecutive test days (*n *= 4 pigs), or if they encountered health problems (umbilical hernia: *n *= 1, lameness: *n *= 1). In this experiment, 51 pigs (48 females and 3 males, and 6 pigs with banana as reward) were successfully trained and included in the analyses. Within one batch, all pigs were tested on the same days.

### Reward downshift

After 11 days, all pigs received a small reward only on another 11 subsequent days (reward downshift, creating a 6–1 vs. 1–1 reward group), i.e. the pigs originally receiving six pieces of apple or banana experienced a reward loss. We planned to proceed to the reward downshift when the pigs in the large reward group would run significantly faster (Burman et al. 2008) than the pigs in the small reward group, but set a maximum of 11 pre-reward downshift days (Burman et al. 2008) to minimise pigs from losing interest in the test. After 11 training days, not all (housing treatment groups of) pigs that received the large reward ran significantly faster than the pigs that received the small reward, therefore the reward downshift started at day 12 and ended on day 22.

### Statistical analyses

SAS (SAS 9.4, SAS Institute Inc.) was used for all statistical analyses.

Censoring occurred for pigs that failed to get the reward within 120 s. Therefore, survival analysis was used for the latency to reward. First, Kaplan–Meier survivor functions were estimated. Subsequently, Cox proportional hazard regression was performed with animal as a random effect to estimate the hazard ratios with 95% confidence interval. The explanatory variables early life housing (pre-housing: B1, E1), current housing (post-housing: B2, E2), original reward size (1, 6), reward downshift (from day 12 onwards, all 6-1 pigs were “YES”; all 1-1 pigs were “NO”), coping style (HR, LR), trial number (1, 2, 3), test day (1, 2,…, 22) and batch (1, 2) were analysed as class variables. A multivariable Cox regression analysis was started with all the variables above and their two- and three-way interactions. Subsequently, a stepwise backward selection procedure was performed deleting non-significant variables starting with the highest *p* value, until all factors in the model had *p* < 0.05. The variables pre-housing, post-housing and original reward size remained in the model, even if they were not significant, as these variables were included in the main research aims in this study. Therefore, the final multivariable model consisted of the variables pre-housing, post-housing and their interaction, reward downshift and the interaction of reward downshift with pre-housing, original reward size, coping style, test day and trial number and the random effect of pig.

It was checked whether the proportional hazard assumption was not violated, i.e. the hazard functions of groups are proportional over time. If a hazard ratio (HR) is smaller than 1, then the probability to eat the reward is smaller compared to the reference class, and if it is larger than 1 the probability is higher. If the value 1 is included in the 95% confidence interval, then there is no difference between groups.

## Results

In total, 3355 records could be used based on 51 pigs on 22 test days, with three trials per day per pig.

**Fig. 2:**
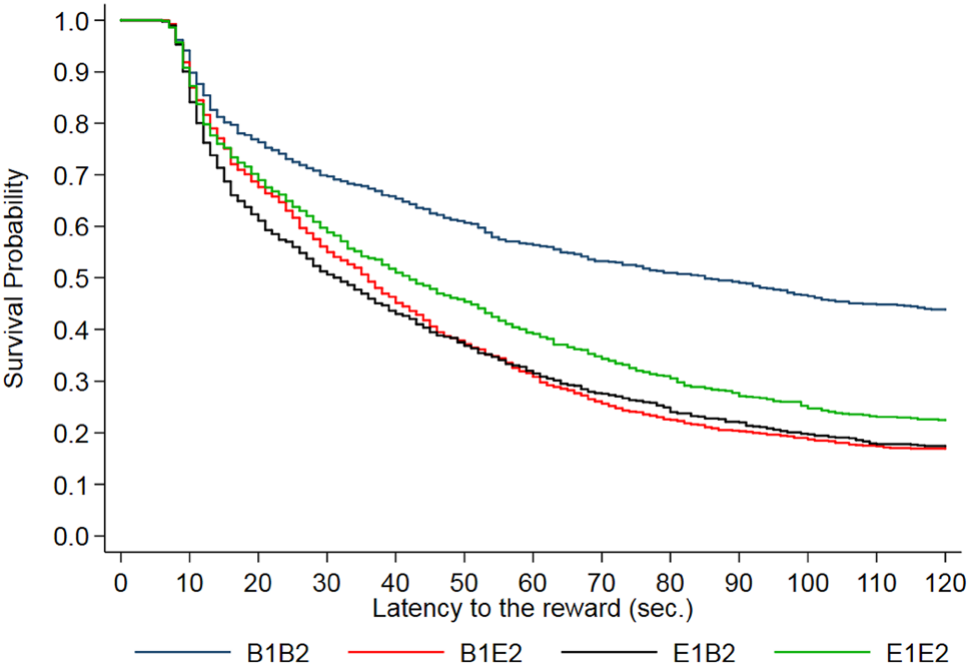
Kaplan-Meier curve showing the latency to reward for pigs from each housing group, with a maximum trial duration of 120 s. For each trial in which a pig got to the reward, the probability on the *Y*-axis drops. B1B2 and E1E2 refer to pigs housed in barren and enriched pens, respectively, throughout the experiment; B1E2 and E1B2 refer to pigs that experienced a change in environment from barren to enriched or vice versa from 7 weeks of age

The probability for trials in which pigs from each housing treatment got the reward at some point in time is illustrated in a Kaplan–Meier curve (Fig. 2, log-rank *p* < 0.0001). The percentage of censored records, i.e. trials in which pigs did not get the reward within the maximum time of 120 s was higher for the B1B2 pigs (43.9%), compared to B1E2 (17.0%), E1B2 (17.4%), and E1E2 (22.5%) pigs.

**Fig. 3:**
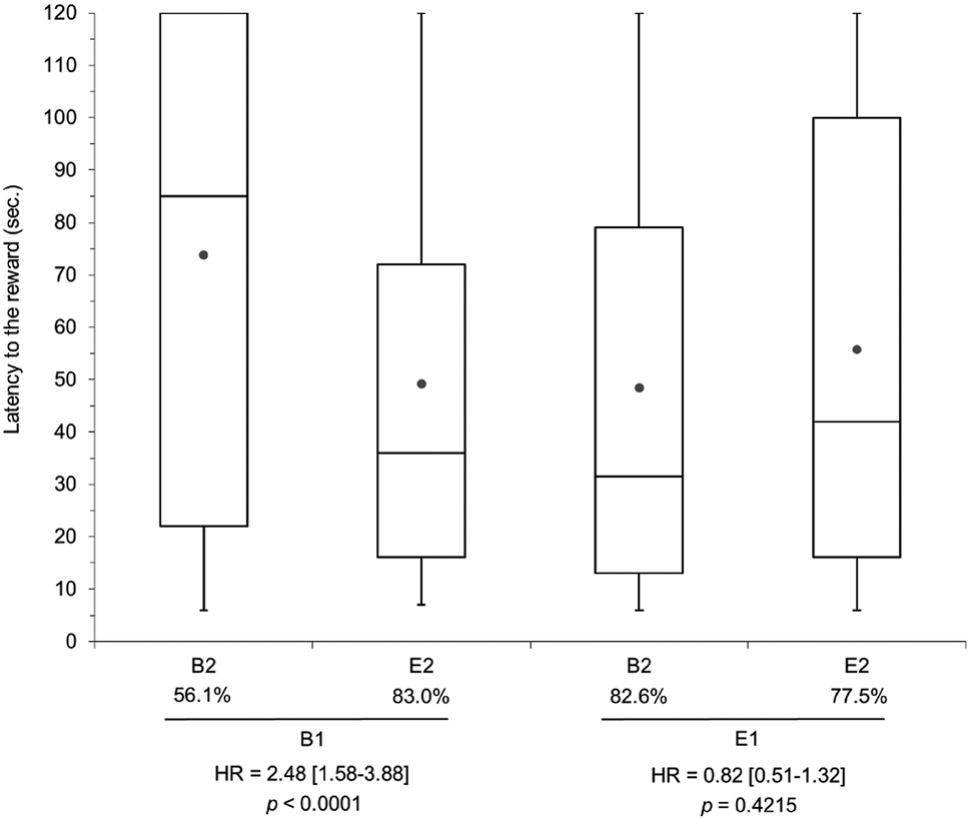
Box-Whisker plot for the latency to the reward with the percentage of total trials to get to the reward (black circle = mean), and the stratum-specific hazard ratio (HR) with 95% confidence interval for the pre-housing × post-housing interaction (*p* < 0.05). B1 and E1 refer to barren and enriched housing in early life, respectively, and B2 and E2 refer to barren and enriched housing from 7 weeks of age onwards. Note that latency to the reward is underestimated because the observations were censored at 120 s

The multivariate model showed that the effect of current housing on latency to the reward depended on the early life housing (pre-housing × post-housing interaction, *p* = 0.001), or vice versa. Pigs that had switched from barren to enriched housing (B1E2) had a higher probability of getting the reward and lower latency to the reward compared to B1B2 pigs housed in barren conditions throughout life (HR 2.48, *p* < 0.0001, Fig. 3); however, for the pigs housed in an enriched environment in early life there was no effect of later life housing (E1B2 vs. E1E2). For barren housed pigs in later life (B2), E1 pigs had a higher probability of getting the reward and a lower latency than B1 pigs, both for no reward downshift (HR 3.61, *p* < 0.0001) and for reward downshift (HR 2.32, *p* = 0.0005). When pigs had enriched housing later in life (E2), there was no effect of pre-housing.

**Fig. 4:**
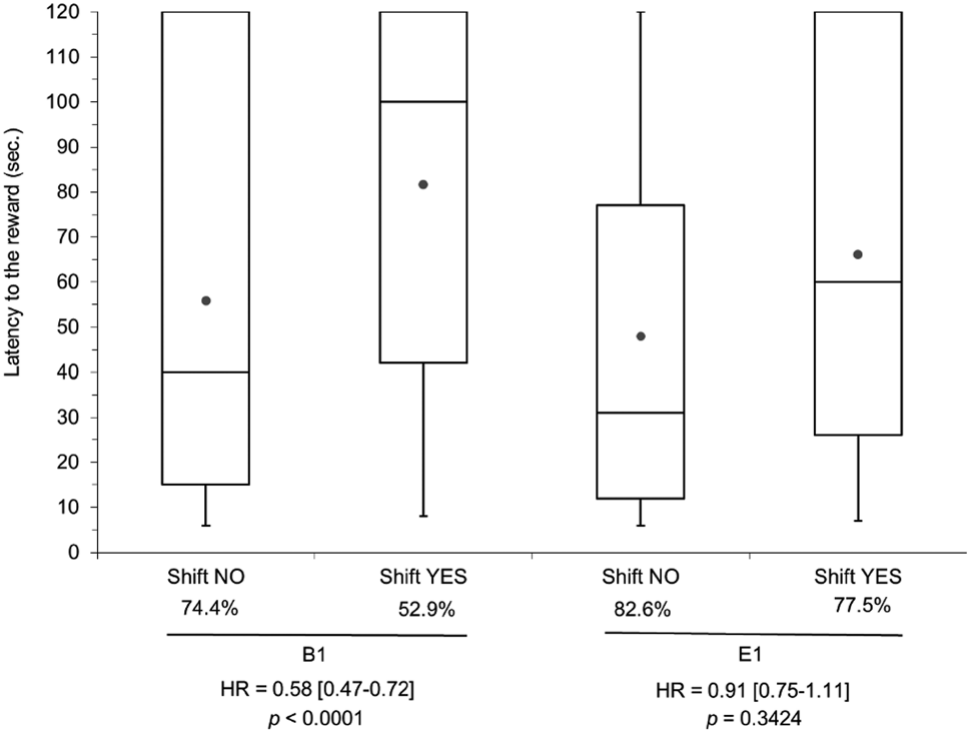
Box-Whisker plot for the latency to the reward with the percentage of total trials to get to the reward, and the stratum-specific Hazard Ratio with 95% confidence interval for the pre-housing × reward downshift (YES or NO) interaction (*p* = 0.0004). B1 and E1 refer to barren and enriched housing in early life, respectively. Note that latency to the reward is underestimated because the observations were censored at 120 s

Latency to the reward was not different between pigs from the large reward group (6–1: 72.8%, 57.1 ± 1.2 s) compared to the pigs that always received a small reward (1–1: 76.2%, 56.0 ± 1.0 s, HR 1.09, *p* = 0.6228) in the whole test period, including the 11 days before reward downshift. Pigs that experienced a reward downshift had a lower probability to get to the reward and a higher latency to the reward, but this effect was only significant for pigs housed in barren conditions in early life (HR 0.58, *p* < 0.0001, Fig. 4) to reflect the interaction between pre-housing and reward downshift (*p* = 0.0004).

**Fig. 5:**
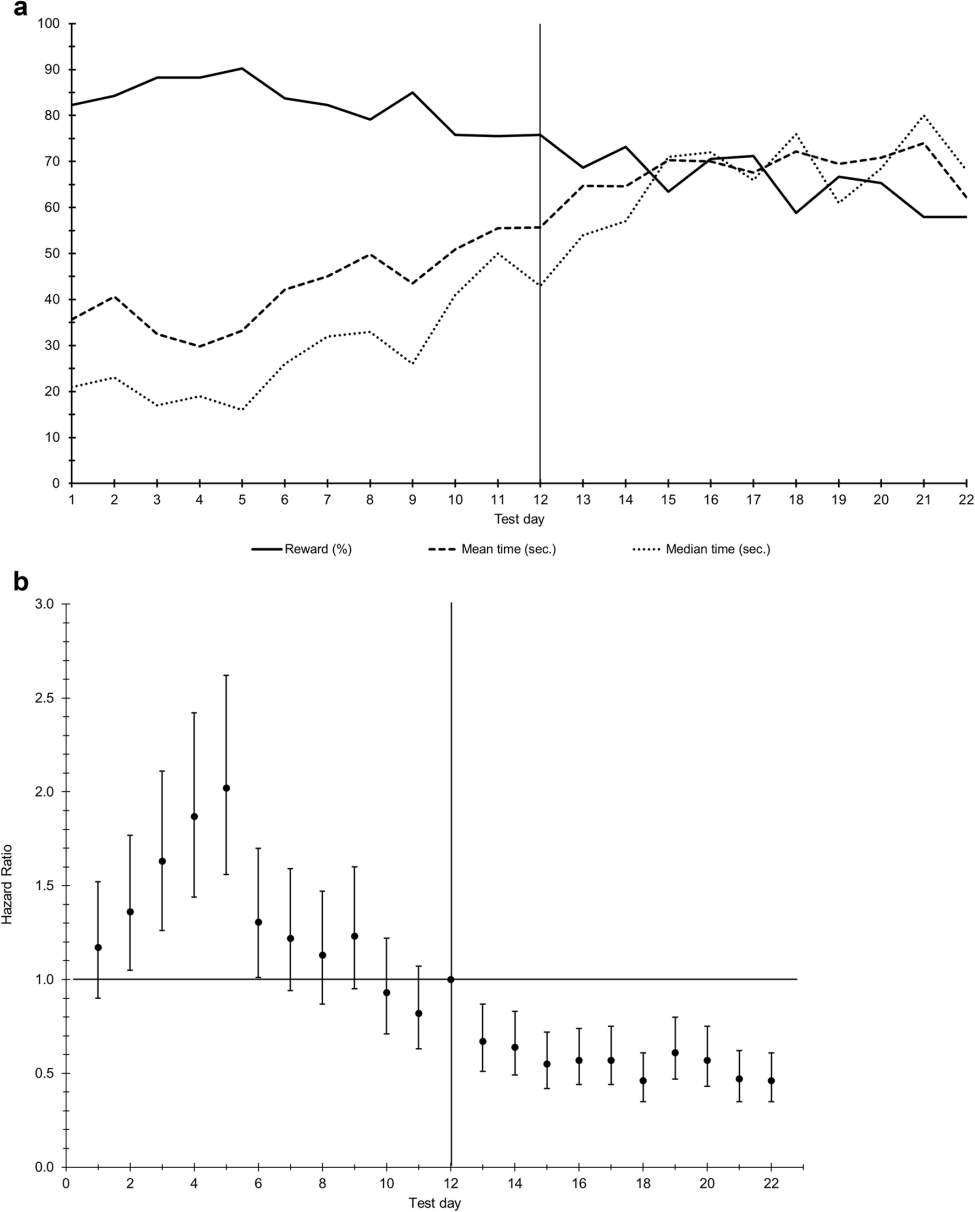
**a** The mean and median time of the latency to the reward and the percentage of total trials in which pigs got to the reward within 120 s for each test day. Note that means and median times are underestimated because the observations were censored at 120 s. **b** Hazard ratios with 95% confidence intervals per test day. If the value 1.00 is within the confidence interval, the hazard ratio is not significantly different from day 12, the day of reward downshift (overall test day effect *p* < 0.0001)

Figure 5a presents the mean and median time of latency to the reward over test days, as well as the percentage of trials in which pigs got to the reward. A test day effect was found (*p* < 0.0001, Fig. 5). At the first six test days, the probability to get to the reward was higher compared to day 12 (the day of reward downshift), being significant for day 2 to day 6 (*p* < 0.05 or less, Fig. 5b). The probability on test day 7–11 was not different from day 12. After reward downshift, the probability to get to the reward decreased and was on all days lower compared to day 12 (Fig. 5b).

Pigs had a lower probability to get to the reward and higher latency to the reward in trial 2 (69.9%, 62.7 ± 1.3 s, HR 0.40, *p* < 0.0001) and trial 3 (66.1 ± 1.2 s, 70.3%, HR 0.35, *p* < 0.0001), compared with trial 1 (84.1%, 39.8 ± 1.2 s) within a test day.

Low-resisters (78.7%, 52.2 ± 1.0 s) had a higher probability of getting the reward and lower latency to the reward compared to high-resisters (71.0%, 60.1 ± 1.0 s, HR 1.48, *p* = 0.0187).

## Discussion

In this study, we aimed to investigate the effect of early and later life housing conditions on the sensitivity to reward loss in pigs in a successive negative contrast test (SNC) using a runway. In the whole test period, both before and after the reward downshift and irrespective of reward size, pigs that were housed barren throughout life had a lower probability and a higher latency to get to the reward than pigs from the other three housing combinations, i.e. enrichment throughout life, enrichment in early life only, or barren housing in early life followed by enrichment. Early life housing affected the sensitivity to reward loss, as only in pigs from early life barren housing (B1E2 and B1B2) an effect of the reward downshift was found. Coping style also affected latency to the reward, with reactive pigs having a higher probability and a shorter latency to get to the reward than proactive pigs.

### Effects of reward loss

Pigs ran faster from day 2 to day 6, compared with day 12 when the reward was downshifted. After the reward downshift, all pigs, including pigs that did and did not experience a reward downshift, ran slower than before the reward downshift and probability to get to the reward reduced. It could be that pigs reduced their interest in the test and reward over time, which is possibly also reflected by the higher latency to the reward in the second and third trial on a test day as compared with the first trial. Alternatively, they may have become slower due to the increase in body weight over test days, as at this age, the pigs gained approximately 1.1 kg per day.

Nevertheless, there was an effect of reward downshift on the latency and probability to the reward, but this effect was only significant in pigs from early life barren housing (see below). When an anticipated food reward is unexpectedly reduced, many mammals show a decreased response to the reward, compared to controls which received only the lower level reward (Bergvall et al. 2007; Catanese et al. 2011). The decrease in reward size may lead to disappointment-like or frustration-like affective responses, which can influence the motivation for the reward (Burman et al. 2008). Thus, even though pigs generally decreased their running speed over time, the reward reduction was still aversive for some of them and caused a successive negative contrast effect in pigs from barren early life housing.

Burman et al. (2008) reported a higher sensitivity to reward loss in rats that experienced a loss of enrichment in their housing environment. In this study, we found that the pigs exposed to barren housing in early life (B1B2 and B1E2) were more sensitive to reward loss, as only in these animals the running speed and probability to get to the reward was affected by the reward downshift. This may suggest a long-term effect of a poor environment on the (negative) affective state in later life.

We expected the highest sensitivity for reward loss in the pigs from early life enriched housing that switched to barren pens in later life, as results of several studies indicate that a loss of enrichment may be more detrimental than no experience with enrichment at all (Beattie et al. 1995; Bolhuis et al. 2006; Douglas et al. 2012). Such an effect was not found, however, as the latency to the reward for E1B2 pigs did not differ from the latency for E1E2 pigs. The reason could be that the appraisal of the test conditions may have interfered with the effects we intended to study, as being (trained) in a test such as a runway may be rewarding in itself and provide cognitive enrichment, thereby inducing a temporary positive affective state. If so, this may have blunted or overruled (early life) housing effects on long-term mood (Bethell et al. 2016; Roelofs et al. 2016). Apart from potential rewarding properties of the test itself, the finding that early life enrichment makes pigs more resilient to reward loss might counteract a potential negative effect of the reduction in environmental quality. Alternatively, results of a recent study suggest that previous exposure to an experience of frustration, which, in our study, could be the case for pigs that switched from enriched to barren pens, may counteract the effects of a new frustrating situation, like SNC (Cuenya et al. 2012). Further research is needed to test such an effect in pigs.

### Motivation to run for the reward

We found that B1B2 pigs, irrespective of reward size, had lower probability and higher latency to get to the reward than the other pigs, both before as well as after the reward downshift, which may show that pigs kept in barren housing conditions throughout life have a lower motivation to run for the reward. Diminished sensitivity to reward can be interpreted as a characteristic of poor mood or anhedonia (Von Frijtag et al. 2000). Anhedonia is the reduced reactivity to pleasurable stimuli or positive effects from events or activities and is one of the core symptoms of depression (Bevins and Besheer 2005; Leppänen 2006; Von Frijtag et al. 2000). Indeed, it has been found that chronic stress caused by tail handling led to a lower response to reward in mice (Clarkson et al. 2018), and juvenile isolation reduced motivation for sucrose drinking in rats (Van den Berg et al. 1999). As a depression-like state or chronic stress has also been linked with barren housing conditions (Beattie et al. 2000; Douglas et al. 2012), the lower probability to get to the reward in the B1B2 pigs in this study may have reflected anhedonia and a low reward sensitivity. On the other hand, some adverse conditions have also been found to increase the sensitivity to reward (Van den Berg et al. 1999). For example, acute stress caused by isolation increased the motivation for food in hens (Hernandez et al. 2015), and chronic mild stress in rats and music inducing a depressed mood in humans increased the motivation for highly positive sweet food rewards, albeit the latter can also be interpreted as a measure of craving rather than response to the reward (Willner et al. 1998). The relationship between mood and reward sensitivity is thus not that straightforward. Moreover, other cognitive studies where pigs had to run to find a food reward as well reported no (Bolhuis et al. 2004, 2013) or only limited (Grimberg-Henrici et al. 2016) evidence for a lower motivation for rewards in pigs when housed barren. Another, alternative, explanation of the lower probability to get to the reward in B1B2 pigs could be that they had spent more time on exploring (parts of) the runway, to ‘catch up’ from the limited space and stimuli in their housing environment. It should be noted, though, that this effect was not seen in barren housed pigs that had been exposed to an enriched environment in early life.

This is, to the best of our knowledge, the first SNC test in pigs. Over pre-shift days, latencies to get to the reward were higher in the last 5 test days. Also, not all pigs that were initially trained were motivated to run for apple pieces, even though apples have been successfully used as a reward in another study which, in addition, demonstrated that pigs prefer four pieces over one piece of apple (Melotti et al. 2013). Motivation could possibly be enlarged in future studies by a longer food deprivation before testing, restricted feeding or by determining the animals’ individual preference for a particular reward before the experiment (Zebunke et al. 2018). It could also be that the pigs in this study lost their interest in the task in general over trials and over test days, so the optimal testing period and number of trials per day need to be considered in future studies.

We did not find a difference in response to the small vs. large reward before the reward downshift, in contrast with a study on rats using 12 vs. 1 food pellets (Burman et al. 2008). However, in another rat study with a similar 1:12 approach (Cuenya et al. 2012), no pre-shift difference in latency to reward was found between the small and large reward group either, and, similar to our study, still an SNC effect could be demonstrated. Unlike previous studies in rats (Burman et al. 2008; Cuenya et al. 2012; Pellegrini et al. 2004) and dogs (Bentosela et al. 2009) which demonstrated a return to pre-shift responses after 5–6 post-shift days, we found no signs of a recovery in running speed in the pigs that experienced a reward loss, as there was no interaction between day and reward group. It should be noted that, even though statistically significant, the effect of reward loss was mild compared to rat studies in which latencies roughly doubled in animals experiencing a reward downshift (Burman et al. 2008; Cuenya et al. 2012). In these rat studies, the ratio between the large and small reward was larger (1:12), and, moreover, the large reward was a substantial part of their daily portion of feed.

Our main interest was to evaluate the effect of rearing and housing conditions on reward loss sensitivity in pigs. As personality traits of animals have an effect on their behavioural responses, including those in tests for affective state (Cuenya et al. 2012; Franklin et al. 2016), we characterised all pigs early in life by the backtest as ‘high-resisters’ or ‘low-resisters’ (Bolhuis et al. 2004; Hessing et al. 1994; Reimert et al. 2014a). The response of pigs in this backtest, which is heritable (Iversen et al. 2017; Velie et al. 2009; Zebunke et al. 2015), reflects their tendency to adopt a proactive (also called active) or reactive (also called passive) coping response (Bolhuis et al. 2005; Koolhaas 2001; Koolhaas et al. 1999). To account for a potential coping style influence, allocation of pigs to the housing treatments and reward groups was balanced for backtest classification, which was subsequently added as a fixed effect in the analyses. There was an effect on the latency to reward, as low-resisters had a lower latency to get to the reward than high-resisters. It is difficult to say whether this effect was due to a difference in how low-resisters and high-resisters valued the reward and were motivated for the task, or reflected a difference in balance between different motivations (e.g. to explore the runway vs. go for the reward immediately). Concerning the latter, the shorter latencies of the low-resister pigs are not in line with general findings that these pigs explore for longer, even in a familiar test room (Jansen et al. 2009). Nonetheless, it is important for future research to keep in mind that personality traits, such as coping style, may affect important read out parameters of tests of affective state. The number of animals included in the task did not allow us to test all potential interactions between coping style with the four rearing and housing combinations and the two reward sizes. For future studies, however, it could be interesting to further investigate potential interactions between environmental conditions and personality, which have been found for judgement bias (Asher et al. 2016), behaviour in the home pen (Bolhuis et al. 2005, 2006) and behaviour in a T-maze (Bolhuis et al. 2004), on the sensitivity to reward loss.

## Conclusions

Barren housed pigs and pigs with a proactive coping personality had a longer latency to get to the reward throughout the test which could either reflect their affective state, or, alternatively, a difference in balance between the motivation to explore the test area and the motivation for the reward. Irrespective of their current housing conditions, pigs originating from a barren pen in early life responded to the reward loss, as reflected in a reduced probability and increased latency to get to the reward following reward downshift, whereas such an effect was not found in pigs originating from enriched pens. This may indicate that negative early life experiences may have a long-term impact on the ability of pigs to cope with reward loss and on affective state. We found no clear evidence for an effect of current housing, nor for an effect of a change in housing (from barren to enriched or vice versa) on reward loss sensitivity.
